# Sodium Salicylate for Toothache

**Published:** 1899-03

**Authors:** 


### Sodium Salicylate for Toothache.

Dr. Frederick C. Coley, in an article on the medical treatment of toothache in a recent number of the *Practitioner*, states that of all medical remedies for toothache he knows of none which is so successful as salicylate of sodium. He believes it is especially useful in those cases where the pain is started by "taking cold."

A dose of gr. xv will usually relieve the pain very promptly, and if this is repeated every four hours the inflammation may entirely subside, leaving, of course, a carious tooth to be disposed of according to circumstances. The addition of belladonna is often advantageous. Fifteen grains of sodium salicylate, with fifteen minims of tr. belladonna will often procure refreshing sleep instead of a night of agony. It is especially valuable with children, where extraction of teeth is to be avoided, if possible, lest the development of the maxilla should be injured.—*N. Y. Med. Times.*

